# Family Member and Healthcare Provider Perceptions of Factors Influencing Undernutrition Among Infants and Young Children in South Asia: A Systematic Review of Qualitative Studies

**DOI:** 10.3390/nu18050776

**Published:** 2026-02-27

**Authors:** Md. Fakhar Uddin, Shariffah Suraya Syed Jamaludin, Harn Shian Boo, Akash Saha, Asma-Ul-Husna Sumi, Tahmeed Ahmed, Judd L. Walson, James A. Berkley, Sassy Molyneux

**Affiliations:** 1 Anthropology & Sociology Section, School of Social Sciences, Universiti Sains Malaysia, Pulau Pinang 11800, Penang, Malaysia; shariffah@usm.my (S.S.S.J.); booharnshian@usm.my (H.S.B.); 2 Nutrition Research Division (NRD), International Centre for Diarrheal Disease Research, Bangladesh (icddr,b), 68, Shaheed Tajuddin Ahmed Sarani, Mohakhali, Dhaka 1212, Bangladesh; akash.saha@icddrb.org (A.S.); asma.sumi@icddrb.org (A.-U.-H.S.); tahmeed@icddrb.org (T.A.); 3 The Childhood Acute Illness and Nutrition (CHAIN) Network, Nairobi 184742, Kenya; jwalson1@jh.edu (J.L.W.); jberkley@kemri-wellcome.org (J.A.B.); smolyneux@kemri-wellcome.org (S.M.); 4 Departments of International Health, Medicine and Pediatrics, Johns Hopkins University, Baltimore, MD 21218, USA; 5 Centre for Tropical Medicine and Global Health, University of Oxford, Old Road Campus, Headington, Oxford OX3 7BN, UK

**Keywords:** perceptions, factors, undernutrition, infants and young children, South Asia

## Abstract

**Background**: Undernutrition among infants and young children in South Asia remains a major public health concern, contributing to high rates of morbidity and mortality. While quantitative systematic reviews have identified various risk factors for undernutrition, no review has focused on qualitative studies. This study aims to review published literature on family member and healthcare provider perceptions about influences on undernutrition among infants and young children in South Asia. **Methods**: We searched for qualitative research articles published from 2000 to 2026 in the PubMed, Scopus and CINAHL databases, and used the Critical Appraisal Skills Program (CASP) tool to assess the quality of selected articles. Selected articles were analyzed thematically. The PROSPERO registration number is CRD42022385382. **Results**: After screening 201 research articles, 19 articles were selected for inclusion in this review. Perceived influences of undernutrition among children were categorized into individual, socio-cultural, economic, environmental and system factors. Interconnected influences included maternal illness, single motherhood, mothers’ knowledge and awareness, convenience of providing low-quality ready-made and junk food, spiritual beliefs and superstition, violence against women, financial constraints in a context of rising food prices and seasonal impacts on food production, and physical accessibility of healthcare services. **Conclusions**: This review emphasizes the complex interplay of influences on undernutrition among young children in South Asia. Potential interventions must be culturally tailored and gender-sensitive, with key strategies including nutrition education, community-based support, maternal health improvements, and policies addressing food insecurity and healthcare accessibility.

## 1. Introduction

Undernutrition refers to a deficiency of energy and nutrients (e.g., vitamins, iodine, zinc, protein) in relation to the requirement for health and growth [1]. Undernutrition remains a pressing global health challenge, disproportionately affecting low-and middle-income countries. The World Health Organization (WHO) identifies three ways to assess undernutrition using anthropometry: wasting (low weight for height), stunting (low height for age) and underweight (low weight for age) [2]. In 2022, globally, 149.2 million (22.3%) and 45.4 million (6.8%) children under five were stunted and wasted, respectively, with South Asia accounting for 53.8 million stunted and 7.6 million wasted children [3]. South Asia bears a disproportionate burden of child undernutrition, accounting for nearly 40% of global stunting and over 50% of global wasting, representing a critical public health problem [4].

Undernourished children have impairments in immunity, delayed motor and cognitive development, blindness, anemia, recurrent infections, impaired behavioral development, and an increased risk of morbidity and mortality [5,6,7,8,9]. Furthermore, early childhood undernutrition impairs adult productivity, increases child birth complications and hinders economic growth, all of which undermine progress towards Millennium Development Goals (MDGs) and Sustainable Development Goals (SDGs) [5,10]. In 2015, South Asia failed to meet nutrition-related MDG targets, despite some improvements in child mortality, and showed inadequate progress due to persistent inequalities in food security, healthcare access, maternal health and education [3,11]. In South Asia, undernutrition among children is an important challenge in achieving multiple SDGs, including health and well-being (SDG 3), zero hunger (SDG 2), and clean water and sanitation (SDG 6) [12]. Addressing child undernutrition is essential, as it directly impacts child morbidity and mortality rates and undermines efforts to eliminate hunger and improve health outcomes in the region.

Undernutrition is a multifactorial issue influenced by a range of socioeconomic, environmental, and health system-related factors across different settings. Studies from low- and middle-income countries have highlighted the role of food insecurity, maternal education, and access to health services as important determinants of child undernutrition [13,14]. In Southeast Asia, the growing influence of climate change, urbanization, and changing dietary practices is shaping malnutrition [15]. Quantitative empirical studies [16,17,18,19,20] in India, Pakistan and Bangladesh have identified factors associated with stunting, wasting and underweight among under-five children. These factors include household wealth (monthly income less than US$42.02), low parental education and occupation, child’s age (24–35 months because of supplementary food) and sex, birth order, low birth weight, unimproved sanitary facilities and sources of drinking water, age of mothers (age less than 20 years), mother’s low BMI (less than 18.5) and diseases (diarrheal episode, infection, and fever), place of residence, religion (Muslim children at higher risk), and caste. In South Asia, gender inequality, poor sanitation, seasonal food insecurity, and overburdened public health systems have also contributed to poor nutritional outcomes [21]. Collectively, these findings emphasize the importance of a nuanced understanding of undernutrition that considers both context-specific drivers and broader structural determinants.

While quantitative studies reveal statistical associations between risk factors and nutritional outcomes, they fail to capture complex lived experiences, cultural beliefs, and contextual nuances shaping undernutrition among children in South Asia. Qualitative research provides critical insights into how and why these associations occur, revealing mechanisms through which structural influences translate into nutritional outcomes at household and community levels. Understanding perspectives from family members and healthcare providers can be a key element of qualitative studies, providing essential information for designing culturally appropriate, contextually relevant interventions.

Despite the wealth of individual qualitative studies conducted across South Asian countries, no systematic synthesis of the findings on family and healthcare worker perceptions exists, representing a missed opportunity to inform policy and practice. Systematic reviews of qualitative evidence can identify common themes across diverse contexts, reveal context-specific variations, and generate more comprehensive insights than an individual paper alone can provide. This review aims to examine published literature on family and healthcare provider perspectives regarding influences on undernutrition among infants and young children in South Asia. By systematically synthesizing qualitative evidence from across the region, this review provides valuable insights for developing culturally sensitive, context-appropriate interventions aimed at mitigating vulnerabilities to undernutrition.

## 2. Materials and Methods

This review systematically analyzed original qualitative and mixed-method studies that examined the influences of undernutrition among young and infant children. This review’s protocol was registered with PROSPERO under the code CRD42022385382, and was conducted following Preferred Reporting Items for Systematic Reviews and Meta-Analyses (PRISMA).

### 2.1. Inclusion and Exclusion Criteria

The inclusion criteria for this systematic review included published qualitative and mixed-method studies from South Asian countries (Bangladesh, India, Pakistan, Nepal, Bhutan, Sri Lanka, and Afghanistan) that reported influences of undernutrition among under-five children from the perspectives of children’s family members and health care providers. The exclusion criteria included studies that only reported quantitative data and studies focused on children older than five years and conducted outside South Asian countries.

### 2.2. Search Strategy and Identification of Papers

**Figure 1:**
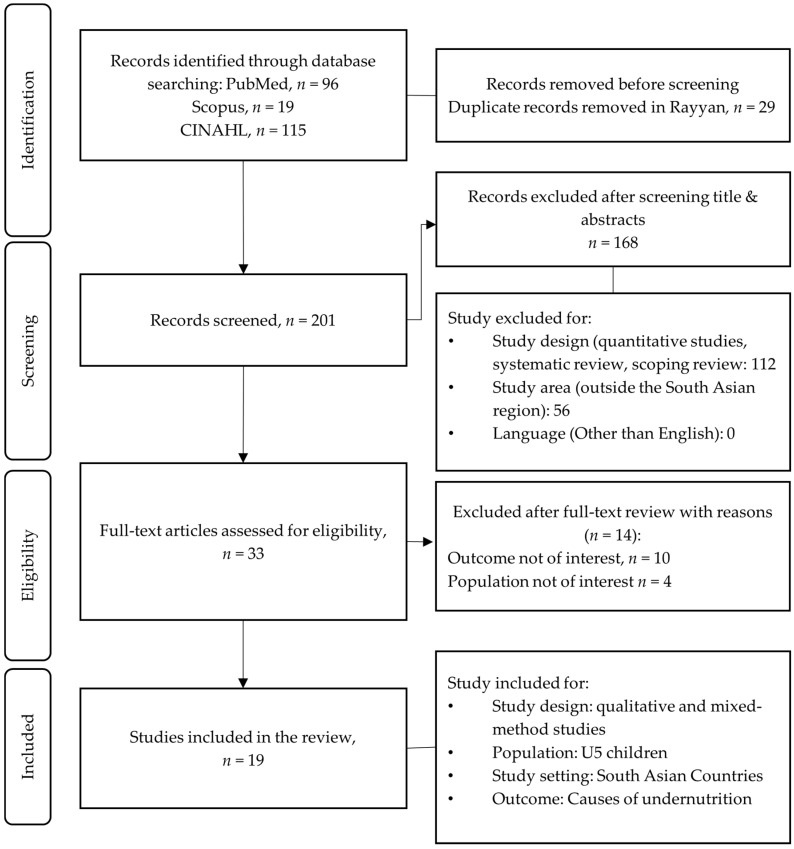
PRISMA flow diagram for study selection with inclusion and exclusion criteria.

This systematic review and interpretative synthesis used a free full-text search strategy in three electronic databases: PubMed, CINAHL and Scopus from January 2000 to January 2026, focusing on published articles in English. The first search was conducted on 26 January 2023 and then repeated on 15 December 2023 using the search strategy and search terms outlined in Supplementary File S1. The final search was completed on 28 January 2026 to ensure inclusion of recent articles. In the initial search, 96 articles from PubMed, 19 articles from Scopus, and 115 articles from the CINAHL database were identified. The keywords used for the search were: [“Caregiver*” OR “Mother” OR “Father” OR “Family member” OR “Community representative” OR “Community member” OR “Community elites” OR “Local leaders” OR “Community health worker”] AND [“Causes*” OR “Factor” OR “Influence” OR “Perception” OR “Perspective” OR “Opinion” OR “views”] AND [“Children undernutrition*”OR “Malnutrition”] AND [“South Asia*” OR “Bangladesh” OR “India” OR “Pakistan” OR “Sri Lanka” OR “Bhutan” OR “Afghanistan” OR “Nepal”] AND [“Qualitative study*” OR “Mixed method study”]. Search results were exported to the Rayyan software to remove duplicates (*n* = 29), and 201 articles were identified for screening. Two researchers independently reviewed the titles and abstracts of the articles to exclude ineligible studies (*n* = 168) and identified 33 articles for a full-text review. After the full-text review, a total of *n* = 19 articles were identified and selected [22,23,24,25,26,27,28,29,30,31,32,33,34,35,36,37,38,39,40] for data synthesis. In cases of disagreement, the two researchers consulted with a third reviewer, and a consensus was reached through discussion. The excluded ineligible articles were recorded in the Rayyan database (Figure 1) with justifications. The cross-references were also reviewed to ensure that no relevant articles were overlooked.

### 2.3. Data Extraction and Analysis

A data extraction form was used for this review, following the process that included pilot testing, refining the data extraction form, and cross-checking by multiple reviewers. Specific data fields were extracted from each selected study. The data field included author, year, study design, setting and type, population characteristics, study objectives, data collection method, data analysis method, key findings and relevant outcomes. Two reviewers independently read and re-read all selected articles to comprehend the findings in relation to the research questions and objectives.

Using an inductive thematic analysis approach, two authors independently performed open coding on four selected articles, allowing codes to emerge directly from the text. After completing the open coding, the two authors met to compile the codes and then discussed discrepancies with the first author to resolve differences and developed an inductive code list. The analysis then proceeded through three distinct steps: first, line-by-line coding of the extracted data; second, categorization of descriptive themes; and third, formulation of analytical themes [41,42,43]. First, one reviewer coded the text in the results sections of the selected articles, focusing specifically on factors of undernutrition among young children according to the code list. A second reviewer then checked each coded segment to ensure accuracy. Next, the codes were organized into descriptive themes by identifying relationships between codes and themes, after which the first author (MFU) developed analytical themes. These themes were reviewed and discussed with the third and fourth authors (AS and AUHS) until consensus on the final analytical themes was reached.

Upon completing the coding of all selected articles based on the analytical themes and sub-themes, a matrix table was created in Microsoft Word with four columns. The columns included analytical themes, sub-themes, coded data, and interpretation. The data coder extracted all the coded data into a matrix table for clarity and systematic analysis. Other authors checked the table to ensure the coded data were correctly placed. Both authors (MFU and AS) then performed an initial interpretation and synthesis of the coded data, followed by a discussion of their interpretations. The third reviewer resolved any differences between reviewers regarding coding, data extraction, and interpretation of findings. In these ways, this review adhered to ethical standards for secondary data analysis, including respect for the original authors’ interpretations and responsible reporting of participant perspectives as presented in the primary studies.

### 2.4. Quality Assessment

The quality of the included studies was evaluated using the Critical Appraisal Skills Programme (CASP) guidelines to assess methodological quality and accuracy [44]. The selected studies were scored ‘Yes’, ‘No’, and ‘Unclear’ following the ten questions of the CASP guideline (Supplementary File S2). Every “Yes” was allocated 1 point, every “No” 0 points, and every “Unclear” 0.5 points. Papers were classified into high-quality papers (scores 7.5–10) and lower-quality papers (less than 7.5). To identify intra-rater reliability, Cohen’s Kappa measures were used [41]. For each paper, the relevant text following the CASP questions was scored by the first (MFU)and fourth author (AS) independently. The screening yielded a Cohen’s kappa (k = 0.83), indicating almost perfect agreement between the two reviewers [45,46]. All the listed articles met the key criteria set in the CASP guideline for high-quality qualitative research. Reviewers identified several concerns regarding the relationship between researchers and participants in some studies, but overall, each paper was deemed valuable, and no papers were excluded based on quality.

## 3. Results

The synthesis encompassed nineteen peer-reviewed qualitative and mixed-method papers from three South Asian countries (Bangladesh, India, and Pakistan) published between 2000 and January 2026. Table 1 displays the characteristics of each included article for the synthesis. Of the nineteen studies, fifteen are exclusively qualitative, and four are mixed methods. Some studies were conducted in challenging environments based on poor human development indicators, higher prevalence of (severe acute malnutrition) SAM among children [29,30], high levels of violence, stress, crime, and drug activity [30], increased population [26], and water scarcity [23]. These studies identified five sets of influences on undernutrition among young children: individual, socio-cultural, economic, environmental and health-system influences. These sets of interrelated factors are described in the following paragraphs.

### 3.1. Individual Influences

#### 3.1.1. Mothers’ Beliefs, Knowledge and Awareness

Mothers, community health workers, and pregnant women in Bangladesh, India, and Pakistan reported that some caregivers lack proper understanding and awareness regarding key childcare practices such as exclusive breastfeeding, preparing nutritious food safely, introducing complementary food at appropriate times, ensuring drinking water safety, maintaining immunizations schedules, and following hygienic protocol (for instance, failing to clean breasts before feeding, leaving drinking water uncovered, or preparing food in unsanitary kitchens) [22,24,25,29,30,33,34,35]. These knowledge gaps contribute to children suffering from recurring illnesses such as diarrhea, scabies, pneumonia, asthma, which eventually exacerbate undernutrition [22,26,29,30,32,35,40]. One mother stated:

*“Lack of awareness… lack of knowledge on nutrition, especially not having knowledge about breastfeeding… could be the main reasons.”*

(A 35-year-old health worker, FGD) [24]

Some mothers were concerned that their breast milk is thin, polluted, or has inadequate nutritional value if they fall sick, which discourages exclusive breastfeeding and leads to the introduction of complementary foods, sometimes within the first three months [22]. The supplementary foods commonly provided include snack preparations that lack vital nutrients. Authors argue that this temporarily satisfies hunger but inadequately supports children’s healthy growth and developmental needs [34].

*“My milk is bad, thin, child cry with this and kicks me; why should I prefer breastmilk, which is no thicker than formula milk, it looks like muddy water (mela pani), which has no power to make my baby fat? Why should I not use formula milk, which is thicker, tasty, and looks like pure milk?”*

(Mother, IDI) [22]

#### 3.1.2. Maternal Illness

Parents and health workers in Bangladesh and India reported that maternal health problems, including sore breasts, headaches, gastrointestinal pain, respiratory infections, poor nutritional status, and anemia, negatively impact mothers’ ability to follow optimal infant feeding recommendations (such as colostrum feeding, exclusive breastfeeding, and timely introduction of complementary foods), affecting their children’s nutritional wellbeing [22,24,25,27,29,30,31,32,33,35,37,38]. According to pregnant women and community health workers, maternal illness reduces physical capacity and physiological well-being among women, resulting in suboptimal caregiving and feeding practices that exacerbate childhood undernutrition [30]. Furthermore, parents felt that mothers with histories of low birth weight outcomes, gestational complications, recurrent childhood morbidities, shorter birth spacing, and adolescent marriage can be vulnerable to perinatal and postnatal illnesses, which perpetuates intergenerational cycles of nutritional deficiency affecting their children [24,32,40].

#### 3.1.3. Single Motherhood

In India and Pakistan, mothers and health workers reported that single mothers, whether divorced or abandoned by their husbands, can experience significant stress and financial burdens, which reduce their capacity to adequately feed and care for their children [22,26,29,30,34]. For example, remarried fathers were often described as contributing less financially to their previous families, placing an increased financial burden on single mothers to provide food for their children, thereby impacting child undernutrition [30]. A single mother stated:

*“Both mother and father are necessary for children’s care. I am a single mother; their father has left us. How can a mother properly take care of and breastfeed [her baby] if there is no peace at home? My breast milk has become dry due to this everyday stress and violence.”*

(Mother, IDI) [22]

#### 3.1.4. Parental Behaviors

Mothers in Bangladesh, India and Pakistan reported that fathers’ alcohol addiction, violence towards mothers, psychological abuse, and sexual coercion led to mental stress in mothers, which reduces their ability to provide adequate care and attention to their children, contributing to undernutrition [22,26,29,30,34,36,38,39]. More generally, practices such as sharing meals on a single plate with siblings, failing to provide special meals for malnourished children, offering ready-made snacks instead of nutritious homemade food, and avoiding breastfeeding to preserve a sexually attractive body image can also contribute to undernutrition [26,29,30]. Health workers in Bangladesh and India note that some mothers are unwilling to use contraception, despite its availability, due to concern that it might adversely affect their physical appearance and sexual attractiveness. This reluctance is reported to increase the number of children and exacerbate the challenging living conditions that contribute to undernutrition [30,37].

*“If the husband is a drunkard, then the mother is too stressed to give attention and care to the children when they are sick.”*

(Mother, IDI, Bihar) [29]

Additionally, mothers’ extramarital relations were seen to reduce their care for their children, contributing to child wasting [38].

### 3.2. Socio-Cultural Influences

#### 3.2.1. Early Marriage

Mothers in Bangladesh and India reported that early marriage often leads to pregnancies before girls are physically ready, resulting in poor nutritional status during pregnancy and post-partum [24,37,40]. This eventually negatively impacts breast milk production and feeding capacity, contributing to undernutrition in their children [24,37]. Moreover, marriage at an early age reduced teenage mothers’ ability to build awareness of how to care for their children [38]. According to mothers, early marriage is influenced by cultural and social factors, including the belief that girls can discontinue their education earlier than boys, as they are not expected to support their families once married [24]. Limited access to nutritious foods for pregnant and lactating mothers affects their ability to produce enough breast milk and properly care for their children.

*“If the mother of the baby is malnourished, then the baby can be malnourished too. The village girls are married off early at an early age. Their body is not fit enough to have the baby and they do not get nutrition properly which leads to a malnourished baby.”*

(A 35-year-old father, FGD) [24]

#### 3.2.2. Spiritual Beliefs and Superstitions

Mothers and mothers-in-law in studies conducted across rural and urban India, Bangladesh, and Pakistan perceived that child undernutrition is often linked to beliefs in supernatural power, such as the evil eye or crossing of a vulture [22,27,28,31,34,40]. For example, some working mothers avoid exclusive breastfeeding due to these beliefs, such as not taking their children to work to prevent exposure to the evil eye [22]. Additionally, some caregivers believed that a child’s wasting was a result of a deity’s wishes [38]. A caregiver expressed:

*“He is Allah who gives it, and He also cures it (Allah diyer de, Allah’r usilay gom oy de).”*

(Caregiver, FGD) [38]

*“My mother stopped me from breastfeeding and asked first to wash my breasts because I was coming from an ill-omened ceremony, which might bring misfortunes for a small baby. I obeyed my mother and acted upon her advice, as it seemed appropriate to me.”*

(Mother, IDI) [22]

In Bangladesh, India, and Pakistan, studies have revealed that infants were often given prelacteal feeds such as sacred or ritual water, honey, sweets, herbs, biscuits, tea, fruit, yogurt, and animal milk, instead of being offered colostrum and exclusive breastfeeding [22,24,26,31,35,37]. These practices were reportedly driven by mothers’ concerns that prolonged breastfeeding could cause diarrhea, constipation, or spoil the child’s blood, ultimately leading to child undernutrition [22,24,26,31,35,37]. Some lactating mothers in India reported avoiding breastfeeding during pregnancy out of fear that it could weaken the unborn child’s legs [22,26]. There were also beliefs reported that breast milk produced during pregnancy was contaminated or poisonous and could make the breastfeeding child ill, and that breastfeeding a baby of the opposite sex to the unborn child was harmful [22,26]. Moreover, food taboos for lactating mothers, especially during the first 40 days after birth, further limited access to nutritious foods like duck eggs, fish, pulses, and potatoes [31,40]. One mother mentioned that she often avoided breastfeeding her child after intercourse until she had cleansed her body, believing that breastfeeding from a “profane” body would harm the child [22], amongst other beliefs.

*“If a wife sleeps with her husband, she cannot breastfeed her baby without taking a bath, as the child eats illegitimate (haram) food, becomes an illegitimate baby (harami). Pregnant women should not continue intercourse after 6 months, otherwise, the child might become a criminal (Jurmi) and tend to commit illegal acts and moral crimes in the future.”*

(Mother18-IDI, Pakistan) [22]

#### 3.2.3. Traditional Practices

Community health workers reported that in India, cultural practices related to breastfeeding, complementary feeding, and hygiene influence inadequate feeding practices and recurrent illness in children, contributing to undernutrition [29,34]. For example, a study in the Baiga tribal community in the Balaghat district of Madhya Pradesh, India, revealed that traditional practices such as open defecation (based on the belief that defecating in a closed area is unhygienic) and poor post-defecation hygiene (such as using leaves, soil, or ashes instead of soap for handwashing and stones or leaves instead of water for wiping) were indirectly linked to child undernutrition [34]. Perhaps of particular interest, many community members did not consider ‘low weight’ a significant health concern in their children, leading to delays in seeking treatment and sometimes severe undernutrition [35]. One mother of a SAM child explained,

*“Only weight is low … It means the (child) is not having any disease.”*

(A SAM child mother, IDI) [35]

#### 3.2.4. Household Influences

According to mothers in India and Bangladesh, their ability to apply child care knowledge is often hindered by the dominating role of mothers-in-law, other elderly family members, and the neighborhood, which contributes to child undernutrition [25]. For instance, mothers were unable to provide special meals for their children because influential family members controlled food purchases. In addition, elder family members introduce unhealthy snacks, but mothers felt they could not object due to their status in the family and fear of disrespecting elders. Mothers often chose honey, ghutti jaggery water, goat milk, cow milk, animal and formula milk, packaged biscuits, chocolates, and solid foods instead of feeding colostrum, practicing exclusive breastfeeding [25], and timely introduction of semi-solid homemade foods after six months [30,35]. These practices were reportedly heavily influenced by family members [29,35], particularly mothers-in-law, and eventually contributed to child undernutrition [26]. Mothers who migrated from villages to urban areas and lived in extended families while engaging in income-generating activities found that limited support from family members, increased household responsibilities, stress from family conflicts, and limited decision-making power disrupted exclusive breastfeeding, complementary feeding, and hygiene practices, eventually contributing to undernutrition in their children [25,33].

*“We all were all living together in one house. My brother-in-law’s 4 children, and all of their wives and all that… it’s a lot of pressure. On top of that, my mother-in-law and father-in-law are old, so they weren’t able to properly take care [of themselves] at that time… I was very stressed.”*

(Mother-IDI, Slum-India) [25]

#### 3.2.5. Gendered Influences and Societal Norms

Many of the above findings should be understood in the context of wider gender relations and societal norms. Studies from South Asia, including Bangladesh, India, and Pakistan, have shown that child undernutrition is closely linked to patriarchal norms and societal expectations [22,24,27,29,30,35,37,40]. In these contexts, mothers often have limited autonomy in decision-making related to reproduction and child care, while the preference for a male child leads to frequent pregnancies and reduced maternal control over child-rearing, which negatively impacts maternal health and contributes to child undernutrition [22,24,27,29,30,35,37]. For instance, in rural India, female children suffer from undernutrition due to societal biases in feeding practices and healthcare seeking [27]. In rural Bangladesh, violence against mothers was identified as a significant factor negatively impacting mental well-being and caregiving abilities, which in turn contributed to severe malnutrition in children [24].

Mothers often bear the sole responsibility for household chores along with child care, which contributes to feeding children foods made from refined flour and low-quality edible oil—often sold without any brand description [34,38]. These foods, devoid of nutritional value, have been described as contributing to children’s developing an addiction to junk food such as Mathari, Matar (salty seeds), biscuits, ice cream, Kurkure, noodles, and chips [26,29]. Such snacks are also given to keep children occupied while busy parents work. Children become reluctant to consume more healthy homemade food, leading to skipping of meals and stomach discomfort, which undermines nutrition [26,29]. Furthermore, the desire for male children among some caregivers reportedly led to short birth intervals, which in turn reduced the care of young children and caused severe wasting [37,38].

*“If the family is not economically well off, even then the child gets Rupee 1 daily. At home, snacks like mathari (made of flour and oil) are prepared and kept for the child as lunch. The child still throws tantrums for getting snacks from the shop.”*

(Mother-in-Law, IDI, Madhya Pradesh) [29]

*“Matar (salty seeds in a commercial pack), biscuits and chips are harmful foods for children. But what to do? Children like them very much. Biscuits are made up of refined flour. When a child eats biscuits regularly, it may cause stomach swelling.”*

(Mother, IDI, Sitapur, India) [26]

### 3.3. Economic Influences

#### 3.3.1. Households’ Financial Constraints

In India, Bangladesh, and Pakistan, community health workers, mothers, fathers and grandmothers reported that household financial hardships result in the provision of poor-quality, low-nutrient, and low-energy food to lactating women, infants and young children [22,24,27,28,29,30,31,33,34,35,37,38,40]. This, in turn, affects mothers’ breastmilk production, leads to inadequate breastfeeding and complementary feeding practices, and delays in seeking medical care, all of which contribute to childhood undernutrition [22,24,27,28,29,30,31,33,34,35,37,38]. Additionally, household financial insecurity forces mothers to engage in labor-intensive tasks such as farming, livestock care, and poultry work to support their families [22,26,29,30,31,33,35]. These activities consume their time and energy, leaving them with limited capacity to provide necessary care and adequate nutrition for their children. As a result, they often leave their young children in the care of older siblings, contributing to undernutrition [22,26,29,30,31,33,35].

A mother shared her experience of having to stop breastfeeding after eight months due to work demands, which she felt resulted in her child’s frequent illnesses and undernutrition [22]. Additionally, in India, family members reported that a lack of stable or regular employment opportunities, as well as underpayment for work, exacerbate household financial constraints, and feed into cycles of debt, food insecurity, feeding norms and undernutrition [25,38]. Employed mothers also reported that the absence of daycare facilities at their workplaces forces them to leave their children at home, which disrupts breastfeeding and complementary feeding practices [33].

#### 3.3.2. Food Price and Household Expenditure

In India, family members reported that the rising costs of both food and non-food items, such as medical care, household goods, education, and transportation, have exacerbated household food insecurity [29]. Parents, particularly in urban areas, reported that the high cost of food in cities makes it challenging to provide their children with a diverse diet, further affecting their nutritional status [33].

#### 3.3.3. Commercial Production and Advertisement of Packaged and Junk Food

Mothers and health workers in India reported that the easy access to commercially produced junk and packaged foods, along with the influence of persuasive television advertisements, has encouraged the early introduction of these products into children’s diets [29,33]. These packaged and commercially produced foods are often favored by children due to their appealing color, flavor, and variety, making them more attractive than healthy, home-cooked options, ultimately leading to unhealthy eating patterns. Mothers in focus group discussions reported that their children frequently consume snacks such as biscuits, instant noodles, and ice cream, which causes them to skip homemade food, further worsening children’s nutritional status [29].

### 3.4. Structural and Environmental Influences

Family members and health workers have reported that repeated illnesses such as diarrhea, respiratory infections, and waterborne diseases are driven by unsafe drinking water, poor ventilation in camps, and inadequate sanitation facilities, particularly in remote and urban areas, which contribute significantly to child undernutrition in India, Pakistan, and Bangladesh [22,30,31,33,34,37,38]. They mentioned that these water sources are often contaminated, visibly polluted with worms, open to the environment, shared with animals, and lacking proper purification. A study conducted in marginalized districts of India found that tribal community mothers often experience physical and mental exhaustion from collecting and boiling water for consumption, particularly during illness or menstruation [23]. This exhaustion limits their water intake, negatively affecting breastmilk production and the frequency of breastfeeding, which in turn contributes to undernutrition in their children [23]. Furthermore, in the Baiga tribal region of India, a shortage of vegetables, infrequent market access (with markets operating only once every ten days), and transportation difficulties prevent families from consistently obtaining essential nutrients, further exacerbating undernutrition among children in the community [34].

During the rainy season, water sources are often polluted as they mix with latrine waste and garbage. Flooding can also disrupt gas supplies, making it difficult to boil water or cook regularly. These conditions lead to poor hygiene, less fresh food, and a higher risk of diseases such as diarrhea, scabies, and pneumonia, all of which contribute to rapid weight loss and worsen child undernutrition [30,31,32,36].

*“We wash hands twice in a day, and in summers only once because there is scarcity of water.”*

(Mother, IDI) [34]

Mothers also reported that child undernutrition is heavily influenced by food insecurity caused by seasonal factors, such as insufficient rainfall, which results in unreliable agricultural outcomes [34]. The reliance on seasonal livelihood opportunities like bamboo cutting, which is available for only three months a year, and collecting forest products such as fruits, leaves, honey, and tubers, along with year-round farming, further worsens food insecurity and contributes to child undernutrition [34].

### 3.5. Health Care Delivery System Influences

Mothers and healthcare providers in India, Bangladesh, and Pakistan reported that some barriers included distance to healthcare centres, long wait times, medicine shortages, and limited awareness of available services, which discourage the use of formal healthcare facilities [22,27,31,33,34,35,36]. These challenges cause families to delay seeking treatment, hinder recovery, and lead to inadequate breastfeeding practices [22,27,31,33,34,35,36]. For instance, parents (Gujrat, India) expressed a strong desire to treat their malnourished children in hospitals, but the unavailability of adequate facilities in the hospital hindered them [35]. In the Baiga community, there was a community-based nutrition management service available, but due to a lack of information about this service within the community, it remained underutilized [34].

A study highlighted that mothers introduced formula milk to their children early on due to a lack of knowledge about breastfeeding and insufficient formal counseling from healthcare providers [22]. The early introduction of cow’s milk is often driven by community advice and financial constraints, compounded by inadequate guidance and counseling from healthcare providers [25]. Moreover, mothers from the Baiga community reported that healthcare providers frequently blamed them for their child’s poor health [34]. This blaming behavior creates mistrust between parents and healthcare providers, discourages timely healthcare-seeking, and can worsen health problems, thereby contributing to undernutrition among children [34].

## 4. Discussion

This systematic review synthesizes and analyzes findings from qualitative studies to present the perceived factors contributing to undernutrition among young children in South Asia. These factors are organized within an analytical framework that encompasses five key domains: individual, sociocultural, economic, environmental, and systemic factors. The following paragraphs elaborate on these factors, with particular attention to maternal knowledge and health, cultural beliefs, gender roles, economic and environmental challenges, and access to healthcare services.

Quantitative studies from Africa and South Asia have identified a lack of nutritional knowledge among mothers, particularly among adolescents, as a significant factor associated with undernutrition among under-five (U5) children [47,48,49,50,51]. Our qualitative systematic review explores how maternal beliefs, knowledge and awareness extend beyond feeding practices to influence child undernutrition in Bangladesh, Pakistan and India. Mothers’ knowledge gaps in nutrition, food safety and nutrient-dense food preparation, water quality, immunizations, and hygiene can contribute—among many other influences—to weakened immunity and recurrent illnesses in their children [22,24,25,27,29,30,31,32,33,34,35]. These gaps impede children’s growth and development, ultimately contributing to undernutrition [52]. The review findings align with qualitative studies from African countries [53,54] demonstrating the global relevance of these influences on child undernutrition. For instance, a study in the Nairobi slums of Kenya found that mothers’ limited understanding of nutrient-dense foods and safe preparation methods contributed to recurrent childhood illnesses and growth faltering [54]. African qualitative studies have also identified fathers’ inadequate knowledge of nutrient-rich foods as a contributing factor to undernutrition [53,55,56], particularly given their decision-making power in some households. Moreover, in African studies, other underlying individual factors such as unplanned pregnancy and paternal drug and gambling addiction were described as contributing to children’s undernutrition. These particular aspects were not explored in detail in the papers included in this review, suggesting that this may be an area for further empirical research in South Asia.

This review’s findings suggest that there is an urgent need for targeted nutrition education interventions focusing on adolescent mothers, fathers and other key family members [21], although these must take into account other socio-economic and structural influences on nutrition, as discussed further below. Education interventions can reduce vulnerability to undernutrition by improving child care practices [57,58,59,60]. Community health workers and peer support groups can play a vital and trusted role in disseminating accurate information and facilitating behavior change [21,61]. Socio-economically and culturally sensitive, family-centred education strategies should be integrated into maternal and child health programs at local, national, and international levels [62].

Quantitative studies in low- and middle-income countries (LMICs) have identified associations between maternal and child undernutrition [49,63]. This review extends this evidence by exploring how maternal physical and mental illnesses specifically disrupt child feeding and caregiving practices in Bangladesh, Pakistan, and India [22,24,25,27,29,30,31,32,33,35,37,38]. This has been documented elsewhere, too, for example, in Kenya, where maternal undernutrition and health problems were found to create a cycle of inadequate caregiving that perpetuated child undernutrition across generations [63,64]. This also aligns with findings from the United States, where maternal illness during lactation was associated with suboptimal breastfeeding practices [65], leading to undernutrition among children. These insights call for a broader focus on maternal health in child nutrition programs and research in South Asia [4]. Potential interventions can prioritize maternal nutrition and well-being through strategies such as providing balanced energy and micronutrient supplements; delivering targeted nutrition education through trained community health workers; strengthening postnatal care; incorporating mental health support into child health programs; and implementing supportive labor policies such as maternity leave and flexible work schedules [4,66,67,68]. Implementing such measures directly supports SDG 3 and aligns with SDG 5 (Gender Equality) by promoting maternal empowerment and equitable health access [14,69]. These approaches can be supported through national maternal and child health guidelines and international frameworks, such as UNICEF’s and WHO’s nutrition action plans [68].

This systematic review reveals that socio-cultural factors significantly hinder child nutrition in South Asian contexts. Two important themes emerged: cultural beliefs and gender dynamics across countries. Existing qualitative studies in low- and middle-income countries have revealed that some cultural beliefs (for example, linked to witchcraft, evil eyes, and taboos around certain foods for lactating mothers and children) can hinder the intake of nutritious foods and delay access to formal health care for children, which can contribute to severe undernutrition [56,70,71]. Supernatural powers, indicated by the crossing of a vulture and through a deity’s will, are believed by some in Bangladesh, India and Pakistan to cause undernutrition in children [22,27,28,31,34]. These deep-rooted cultural beliefs can restrict optimal breastfeeding practices, the intake of essential nutrients, and timely responses to illness in children [72,73]. Addressing these beliefs is challenging but could assist not only in improving nutritional outcomes but also in advancing SDG 2 (Zero Hunger) and SDG 3 (Good Health and Well-being) [74]. To achieve these goals, culturally sensitive interventions that respect traditions while promoting evidence-based practices are needed [56,75], ideally complementing interventions targeting deeper structural drivers (such as access to basic services and income). Potential strategies could include community-based nutrition education programs integrating local beliefs with scientific knowledge; collaboration with local influencers such as relevant healers, religious leaders, and respected elders; and culturally appropriate messaging using local languages, symbols, and examples [76,77]. Involving families and community members in the program design can ensure relevance and acceptance [68,77]. These strategies may help address SDGs and align with SDG 17 (partnerships for the goals) by fostering multi-stakeholder collaboration [78]. Broader social disruptions, such as violation of social marital norms and separation of couples and between couples and their family members, can be linked to issues of children’s undernutrition, but were not explored in the included papers.

Patriarchal structures influence undernutrition among children across the region. Mothers face limited household resource access, unequal food distribution, sole childcare responsibilities, and restrictive decision-making autonomy—issues that were also identified in qualitative studies from Kenya, Indonesia, and Malawi [70,79,80,81]. This review identified that patriarchal norms and violence against women influence undernutrition in South Asia as they do elsewhere [22,24,27,29,30,35,37]. In these studies, gender-related influences of undernutrition emerged spontaneously, suggesting the need to conduct dedicated studies using a gender lens. Notably, in marginalized districts in Pakistan, the intersection of extreme poverty, water scarcity, and conservative gender norms appeared particularly pronounced [82]. Significant maternal labor is needed for water collection, which negatively impacts breastfeeding capacity and frequency [23]. This may be exacerbated by mothers having little agency over maternal reproductive decisions and a strong preference for male children, driving short birth intervals [83]. Overall, these findings highlight critical knowledge gaps for future research regarding intersectional gender-related influences on undernutrition among children in the South Asian context.

Quantitative studies in Pakistan and Bangladesh have found a higher prevalence of childhood undernutrition among girls than boys [18,84]. Our synthesis suggested that these differences may reflect societal biases in feeding practices and healthcare-seeking behavior [22,24,27,29,30,35,37]. These gender-based disparities emphasize the importance of formulating guidelines that recognize and address gender nuances [85]. Gender-sensitive nutrition interventions that empower women through promoting equitable resource distribution and decision-making authority can substantially improve children’s nutritional outcomes [79,86,87]. These measures are also crucial for meeting SDG 2, SDG 3, SDG 5 and SDG 10, as they address the root causes of nutritional inequities [88] by empowering women and promoting equitable intra-household resource distribution [14,89].

This review underscores that household financial constraints and food price volatility significantly reduce access to nutritious food, thereby increasing undernutrition among children [54,70,90,91,92]. Economic hardship not only restricts dietary intake but also limits healthcare access and treatment adherence, delaying recovery and worsening nutritional status [22,24,27,28,29,30,31,33,34,35]. This has also been observed in Africa [54,93], supporting international calls (such as UNICEF’s Nutrition Strategy and WHO Global Nutrition Policy Review) to scale up social protection mechanisms, including food provision and cash transfer programs [94,95,96]. However, wider context-specific economic factors, such as sudden increases in government taxes, extortion, high profit margins on low-quality food and medicine, and marketing strategies for low-quality food, can also contribute to a family’s financial burden and lead to the purchase of low-quality foods linked to undernutrition. If and how nutrition strategies can engage with and influence these wider contexts requires careful examination.

Related to the wider context are also environmental influences, including inadequate water and sanitation, overcrowding, pollution, and seasonal disruptions such as flash floods, with important implications for child undernutrition [47,97]. This review identified that seasonal food insecurity, disruption of cooking facilities, and lack of safe drinking water and sanitation challenges exacerbated undernutrition during certain periods in Bangladesh, Pakistan and India [22,30,31,33,34]. In Bangladesh, recurring flooding in low-lying areas disrupts cooking facilities, contaminates water sources, and increases waterborne diseases, creating distinct seasonal patterns of undernutrition not as prominently featured in Indian or Pakistani studies. Additionally, the influence of forcibly displaced Myanmar nationals and refugee populations in Bangladesh [38] represents a unique contextual factor absent from other countries in this review. Moreover, factors such as household environment and rapid weather pattern changes were not addressed in the selected papers. Addressing these environmental determinants is essential for achieving SDG 6 (Clean Water and Sanitation) and SDG 13 (Climate Action). Potential interventions include seasonal clean water distribution, context-specific food aid, supplementary feeding programs, and infrastructure improvements [98,99]. These interventions could be integrated into national nutrition strategies or into other sector initiatives, with the aim of protecting vulnerable populations year-round.

In Sub-Saharan Africa and other LMICs, health system influences on undernutrition among young children included long waiting times, medicine shortages, inadequate child assessment, unprofessional staff behavior, and high costs [100,101,102,103]. Our synthesis reveals similar challenges and influences [22,27,31,33,34,35]. Strengthening primary healthcare infrastructure, ensuring continuous training for health providers, and integrating nutrition counseling into routine care should improve feeding practices and early illness management, and also improve nutritional status [104]. Future research should explore other health system influences, including community-level health worker acceptability and capacity, pediatrician presence in hospitals, and nutritional service availability.

### Strengths and Limitations of the Systematic Review

This systematic review has some strengths. By including qualitative and mixed-method studies from across South Asia, it captures diverse perspectives from those most directly affected by undernutrition in infants and young children. The findings complement quantitative evidence, providing a more in-depth understanding of the range of influences that need to be considered to design contextually appropriate interventions. Study quality was assessed using the CASP framework, and a comprehensive search strategy ensured the inclusion of up-to-date peer-reviewed publications. Thematic analysis provided an overview of influences and identified a number of directions for future research.

This review has several important limitations. First, it did not explore how the different influences interplay to influence undernutrition. Also, although it presents insights into the “what” and “how” questions about determinants, it does not address “how much” each factor contributes. Integrating these qualitative findings with quantitative evidence through mixed-methods systematic reviews or meta-aggregation approaches would provide a more comprehensive understanding.

Second, although the main themes resonate with a broad range of contexts, some of the details are specific to the contexts studied in the papers included. The ways in which different influences come together will be context-specific, and so readers should apply these findings cautiously and consider local adaptation.

Third, significant geographical limitations exist in the papers included. Despite comprehensive searches across all seven South Asian countries, the included studies came from only three countries (Bangladesh, India, and Pakistan), with no qualifying studies from Afghanistan, Nepal, Bhutan, or Sri Lanka.

Language bias may also have influenced our findings. By including only English-language publications, this review has excluded relevant qualitative studies published in regional languages such as Hindi, Urdu, Bengali, or Nepali. These excluded studies might provide different perspectives, particularly on culturally sensitive topics.

Finally, the relatively small number of included studies (19) may not capture the full diversity of research or experiences across South Asia’s heterogeneous populations. Different types of publications (for example, grey literature, books) and different sub-populations (religious minorities, specific tribal groups, migrant communities) may have distinct experiences not included in this review.

## 5. Conclusions

This review highlights the multifaceted factors contributing to undernutrition among young children in South Asia, emphasizing the need for context-sensitive, multi-level interventions. Key influences include maternal knowledge, socio-cultural norms, gender roles, economic challenges, environmental conditions, and healthcare access. The findings emphasize the importance of improving maternal education, addressing cultural and gender related barriers, and improving healthcare access and economic support. Potential effective interventions include nutrition education, community support programs, maternal health initiatives, and policies tackling food insecurity and healthcare inequality. Culturally sensitive, gender-responsive strategies are important for addressing deep-rooted social and systemic issues. Effective collaboration among healthcare providers, policymakers, researchers, non-governmental organizations, and local communities is essential for designing sustainable solutions that improve child nutrition across the region.

The review findings have significant implications for achieving several SDGs, particularly SDG 2, SDG 3, and SDG 5. They emphasize the need for integrated, gender-sensitive, and culturally appropriate policies at both national and international levels. Policymakers and development partners must prioritize multi-sectoral strategies that address the socio-cultural, economic, environmental, and healthcare system factors driving undernutrition in order to accelerate progress toward global nutrition and health targets.

## Figures and Tables

**Table 1:** Characteristics of selected studies.

Study	Country	Study Type	Data Collection Method	Data Analysis Method	Aim of the Study
Sethuraman et al., 2006 [39]	India	Mixed methods	148 semi-structured interviews with women of childbearing age (15 to 49), 19 focus-group discussions (FGDs) with older women, and 11 FGDs with men	Not clearly mentioned	To explore the relationship between women’s empowerment, maternal nutritional status, and the nutritional status of their children aged 6 to 24 months in rural and tribal communities.
Goudet et al., 2011 [30]	Bangladesh	Qualitative	3 focus groups (FGs) with new community health workers, 5 FGs with experienced community health workers, and 2 FGs with pregnant women	Thematic analysis	To examine the root causes of malnutrition as perceived by pregnant women and CHW.
Burtscher et al., 2015 [27]	India	Qualitative	44 key informant interviews (KIIs) with mothers and health workers, 5 FGDs with women, and 9 group discussions (GDs) with men and women	Content analysis	To understand community perceptions of childhood undernutrition, the CMAM programme, and how these affected health-seeking behaviors.
Chaturvedi et al., 2016 [29]	India	Qualitative	384 in-depth interviews (IDIs) with mothers of nourished and undernourished children; 66 FGDs with mothers, grandmothers, fathers, Anganwadi workers (AWW), auxiliary nurse midwife (ANM) and mothers-in-law; 72 non-formal interactions with mothers, grandmothers, fathers, AWW, ANM and doctors	Thematic analysis	To map the perceptions of mothers and other key stakeholders, and to identify emerging drivers of childhood under-nutrition.
Arafat et al., 2018 [24]	Bangladesh	Qualitative	5 FGDs with 29 caregivers, 4 FGDs with 29 health care workers, and 4 KIIs with community leaders and health supervisors	Thematic analysis	To inform and shape future management strategies by understanding caregivers’ and different stakeholders’ perceptions of malnutrition among infants under six months regarding barriers/facilitators to future CBC.
Hossain et al., 2018 [32]	Bangladesh	Qualitative	6 FGDs with mothers, alternate caregivers, fathers, and parental grandmothers in the urban setting; and 6 FGDs with mothers, alternate caregivers, fathers and parental grandmothers in the rural setting	Constant comparative method	To understand caregivers’ perceptions of children’s linear growth and to identify the cultural meanings and perceptions of risk associated with poor height attainment.
Hoq et al., 2019 [31]	Bangladesh	Mixed methods	18 IDIs with caregivers of children aged under five years, 6 FGDs with 42 women, and an informal group discussion with 15 people residing in the study area	Thematic analysis and logistic regression analysis	To identify the risk factors of acute malnutrition among children aged 6–59 months.
Awasthi et al., 2019 [26]	India	Focused ethnography	12 IDIs with 12 mothers of malnourished children < 24 months of age; 6 FGDs with community health workers; 24 IDIs with mothers of well-nourished (WN) children	Thematic analysis	To understand how the community perceived malnutrition and how the child‘s size at birth, infant and young child feeding-related behaviors and the child‘s illness were associated with a decline in the child‘s growth or health.
Chaand et al., 2019 [28]	India	Anthropological	7 Natural group discussions with tribal mothers and non-tribal mothers, 59 IDIs with mothers, social health activists, ANMs, AWWs, community representatives, and observation in 8 different ambulatory therapeutic feeding centre units	Thematic analysis	To investigate the knowledge, perception and practices related to health, nutrition, care practices, and their effect on nutrition health-seeking behavior.
Shirisha, 2019 [34]	India	Qualitative	20 IDIs with mothers of SAM and MAM children, Anganwadi workers, Integrated Child Development Scheme supervisors, Accredited Social Health Activists, public distribution system shopkeeper and registered medical practitioners; 7 formal interviews with public servants; 5 formal interviews with AWWs	Thematic analysis	To understand the contextual factors for Baiga tribal children’s inferior nutritional status.
Ravindranath et al., 2019 [33]	India	Cross-sectional and mixed methods	35 IDIs with pregnant women and lactating mothers; 2 FGDs with men, construction union leaders, staff of non- profit organizations, maternal health activists, doctors, academic researchers working on health or migration, government employees; and participant observation field sites to study children’s interaction with their environment and document various aspects of their everyday lives	Not clearly mentioned	To categorize the current nutritional status of children under the age of five and determine the underlying causes of poor nutritional outcomes.
Athavale et al., 2020 [25]	India	Qualitative	33 IDIs with mothers and paternal grandmothers and 6 home observations	Thematic analysis	To use qualitative methods to assess the underlying barriers and facilitators for caregivers to implement recommended infant and toddler feeding practices in two urban communities in Mumbai, India, to augment existing IYCF-based interventions and help design sustainable and feasible interventional change.
Ahmed et al., 2021 [23]	Pakistan	Qualitative	3 FGDs with community females, community males, lady health workers, and 5 KIIs with males and females	Thematic analysis	To explore household water insecurity experiences and their association with optimal health and nutrition of women and children in the Rajanpur district of Punjab Province.
(Umallawala et al., 2022 [35]	India	Focused ethnography	In-depth observations of 60 families in a home food environment for three consecutive days	Thematic analysis	To explore community-level determinants of malnutrition among malnourished and well-nourished children.
Ahmed et al., 2022 [22]	Pakistan	Qualitative	20 IDIs with lactating mothers	Thematic analysis	To explore infant and young child feeding (IYCF) and deconstruct breastfeeding barriers in mothers of severely malnourished children in one of the most marginalized districts of Punjab province in Pakistan.
Ulahannan et al., 2023 [36]	India	Mixed methods	12 IDIs with primary caregivers, community representatives, frontline service providers, and non-Adivasi persons	Case study	To understand the complex interaction of structural inequalities, co-occurring health conditions, and child under-nutrition among the Adivasi population in North Kerala, India
Manivannan et al., 2023 [37]	India	Qualitative study	47 IDIs with healthcare providers and mothers	Framework analysis	To explore perceptions of healthcare providers and mothers of children with severe wasting on the perceived reasons for severe wasting, constraints on the management and barriers to caregiving and care-seeking practices.
Rahman et al., 2024 [38]	Bangladesh	Qualitative study	13 FGDs and 17 IDIs with the caregivers of the children of 6–59 months, and 8 KIIs with CHCPs from host communities, community leaders from the FDMN communities, physicians, and outreach and site-supervisors from the INFs in the camps	Content analysis	To explore community perception and understanding of access and utilization of services for the wasted children among the FDMN and their nearest host communities.
Alim et al., 2025 [40]	Bangladesh	Qualitative study	30 FGDs with parents, 28 KIIs with healthcare providers, and 16 KIIs with policymakers	Thematic analysis	To explore the perceptions of acute malnutrition‘s underlying factors and consequences among parents, healthcare providers, and policymakers, alongside parents‘ care-seeking behaviors for under-five children with acute malnutrition.

## Data Availability

The original contributions presented in this study are included in the article. Further inquiries can be directed to the corresponding author.

## Supplementary Materials

The following supporting information can be downloaded at: https://www.mdpi.com/article/10.3390/nu18050776/s1, Supplementary File S1: Describes Boolean operator for the literature search for PubMed, CINAHL and Scopus; Supplementary File S2: Critical Appraisal Skills Programme Checklist.

## Declarations

This paper has been prepared in partial fulfillment of the requirements for the Doctor of Philosophy (PhD) degree of the first author (MFU).

## Abbreviations

The following abbreviations are used in this manuscript:

AWW	Anganwadi Workers
ANM	Auxiliary Nurse Midwife
CASP	Critical Appraisal Skills Program
CHWs	Community Health Workers
FDMN	Forcibly Displaced Myanmar Nationals
FGs	Focus Groups
FGDs	Focus Group Discussions
IDIs	In-depth Interviews
IYCF	Infant Young Child Feeding
KIIs	Key Informant Interviews
LMICs	Low- and Middle-Income Countries
MDGs	Millennium Development Goals
PRISMA	Preferred Reporting Items for Systematic Reviews and Meta-Analyses
SAM	Severe Acute Malnutrition
U5	Under-Five Children
WN	Well-Nourished
WHO	World Health Organization
