# Supplementary material for: Family Member and Healthcare Provider Perceptions of Factors Influencing Undernutrition Among Infants and Young Children in South Asia: A Systematic Review of Qualitative Studies
Source: Nutrients. 2026 Feb 27;18(5):776. doi: 10.3390/nu18050776 (PMC12986657; doi:10.3390/nu18050776)
Supplement: Supplementary file 1 [file nutrients-18-00776-s001.zip › nutrients-4109753-Supplementary file 1.pdf]

Supplementary 1: Describes Boolean operator for the literature search for PubMed, CINAHL and Scopus

For PubMed

(((((((((((Caregiver) OR (Mother)) OR (Father)) OR (Family member)) OR (Community representative)) OR (Community member)) OR (Community elites)) OR (Local leaders)) OR (Community health worker)) AND (((((((Causes) OR (Factor)) OR (Influence)) OR (Perception)) OR (Perspective)) OR (Opinion)) OR (views))) AND (Children undernutrition)) AND ((((((((South Asia) OR (Bangladesh)) OR (India)) OR (Pakistan)) OR (Sri Lanka)) OR (Bhutan)) OR (Afghanistan)) OR (Nepal))) AND ((Qualitative study) OR (Mixed method study))

For CINAHL

(young children) OR infants OR toddlers OR preschoolers OR (children under five) OR (pediatric population) OR (Child Preschool) OR Infant AND (risk factors) OR determinants OR predictors OR causes OR Factors OR Influences OR (socioeconomic factors) OR (cultural factors) OR (maternal education) OR (household income) OR poverty OR (food insecurity) OR (dietary practices) OR breastfeeding OR (complementary feeding) OR sanitation OR hygiene OR (healthcare access) OR (maternal nutrition) OR infection OR (diarrheal diseases) OR (parasitic infections) OR (childcare practices) OR (family size) OR (birth order) OR (Maternal Behavior) OR (Breast Feeding) OR (Food Security) OR (Health Services Accessibility) OR Sanitation OR Infection AND undernutrition OR malnutrition OR (Severe Acute Malnutrition) OR (Moderate Acute Malnutrition) OR protein-energy malnutrition OR stunting OR wasting OR underweight OR (growth faltering) OR (anthropometric measures) OR (nutritional status) OR (micronutrient deficiencies) OR (iron deficiency) OR (vitamin A deficiency) OR (zinc deficiency) OR Malnutrition OR (Child Nutrition Disorders) OR (Growth Disorders) OR Thinness AND Caregiver OR Mother OR Father OR (Family member) OR (Community representative) OR (Community member) OR (Community elites) OR (Local leaders) OR (Community health worker) OR (Health worker) AND Perception OR Perspective OR Opinion OR Insight OR viewpoint AND (Qualitative study) OR (Mixed method study) AND (South Asia) OR India OR Pakistan OR Bangladesh OR Nepal OR (Sri Lanka) OR Bhutan OR Maldives OR (Asia Southern) OR (Developing Countries)

For Scopus

TITLE-ABS-KEY (("young children" OR infant* OR toddler* OR preschool* OR "children under five" OR "under-five" OR pediatric*) AND ("risk factor*" OR determinant* OR predictor* OR cause* OR influence* OR socioeconomic OR cultural OR "maternal education" OR "household income" OR poverty OR "food insecurity" OR "dietary practice*" OR breastfeeding OR "complementary feeding" OR sanitation OR hygiene OR "healthcare access" OR "maternal nutrition" OR infection* OR diarrhea* OR parasitic* OR "childcare practice*" OR "family size" OR "birth order") AND (undernutrition OR malnutrition OR "severe acute malnutrition" OR "moderate acute malnutrition" OR "protein-energy malnutrition" OR stunting OR wasting OR underweight OR "growth faltering" OR "nutritional status" OR "micronutrient deficiency*" OR "iron deficiency" OR "vitamin A deficiency" OR "zinc deficiency" OR thinness) AND (caregiver* OR mother* OR father* OR "family member*" OR "community member*" OR "community leader*"OR "community health worker*" OR "health worker*") AND (perception* OR perspective* OR opinion* OR insight* OR viewpoint*) AND ("qualitative stud*" OR "mixed method*") AND ("South Asia" OR India OR Pakistan OR Bangladesh OR Nepal OR "Sri Lanka" OR Bhutan OR Maldives OR "Southern Asia"))
