# Supplementary material for: Family Member and Healthcare Provider Perceptions of Factors Influencing Undernutrition Among Infants and Young Children in South Asia: A Systematic Review of Qualitative Studies
Source: Nutrients. 2026 Feb 27;18(5):776. doi: 10.3390/nu18050776 (PMC12986657; doi:10.3390/nu18050776)
Supplement: Supplementary file 1 [file nutrients-18-00776-s001.zip › nutrients-4109753-Supplementary file 2.pdf]

Supplementary 2: Critical Appraisal Skills Programme Checklist

Table 2- Critical Appraisal Skills Programme Checklist

Question	Sethuraman et al. 2006	Goudet, et al. 2011	Burtscher et al. 2015	Chaturvedi et. al. 2016	Arafat, et al. 2018	Hossain et al., 2018	Hoq, et al. 2019	Awasthi, et al. 2019	Chaand, et al., 2019	Shirisha, 2019	Ravindranath et al..2019	Athavale, et al., 2020	Ahmed et. al. 2021	Umallawala et. al. 2022	Ahmed et. al. 2022	Ulahannan et al 2023	Manivannan MM, et al.2023	Rahman et al. 2024	Alim et al.2025	
Was there a clear statement of the aims of the research?	√	√	√	√	√	√	√	√	√	√	√	√	√	√	√	√	√	√	√	Yes
																				No
																				unclear
Is a qualitative methodology appropriate?	√	√	√	√	√	√	√	√	√	√	√	√	√	√	√	√	√	√	√	Yes
																				No
																				unclear
Was the research design appropriate to address the aims of the research?	√	√	√	√	√	√	√	√	√	√	√	√	√	√	√	√	√	√	√	Yes
																				No
																				unclear
Was the recruitment strategy appropriate to the aims of the research?	√	√	√	√	√	√	√	√	√	√	√	√	√	√	√	√	√	√	√	Yes
																				No
																				unclear
Was the data collected in a way that addressed the research issue?	√	√	√	√	√	√	√	√	√	√	√	√	√	√	√	√	√	√	√	Yes
																				No
																				unclear
Has the relationship between researcher and participants been adequately considered?	√		√		√	√				√	√						√	√		Yes
							√	√				√	√							No
		√		√					√					√	√	√			√	unclear
Have ethical issues been taken into consideration?	√	√	√	√	√	√	√	√	√	√	√	√	√	√	√	√	√	√	√	Yes
																				No
																				unclear
Was the data analysis sufficiently rigorous?		√	√	√	√	√	√	√	√	√	√	√	√	√	√	√	√		√	Yes
																				No
	√																			unclear
Is there a clear statement of findings?		√	√	√	√	√	√	√	√	√	√	√	√	√	√	√	√	√	√	Yes
																				No
	√																			unclear
How valuable is the research?	√	√	√	√	√	√	√	√	√	√	√	√	√	√	√	√	√	√	√	Yes
																				No
																				unclear
